# Utilizing data to foster equity in infection prevention outreach among skilled nursing facilities in Michigan

**DOI:** 10.1017/ash.2023.295

**Published:** 2023-09-29

**Authors:** Christine White, Michael David, Ruben Juarez

## Abstract

**Background:** Since October 2020, the Infection Prevention Resource and Assessment Team (IPRAT) has provided infection prevention guidance and support to congregate-care settings throughout Michigan. Specifically, outreach to skilled nursing facilities (SNFs) in response to reported positive COVID-19 resident and staff cases. Case rates provide limited data and do not factor in additional variables, such as staffing shortages, geographical location, or access to supplies, which can increase the vulnerability of staff and residents to outbreaks. To facilitate equitable outreach, a risk assessment was developed using variables related to infection prevention and poor COVID-19 outcomes utilizing local, state, and federal data reporting websites. **Methods:** A retrospective data review of IPRAT’s electronic data repository was performed, and 2 distinct periods were identified between November 6, 2020, and December 5, 2022. Outreach method 1 involved only using case counts from November 6, 2020, to September 24, 2021. Outreach method 2 (new risk-assessment–based outreach) involved additional data points from April 12, 2021, to December 5, 2022. Data included 17 self-reported items from the NHSN, 3 characteristics regarding facilities’ COVID-19 units, and 7 community-level variables derived from county vaccine rates, social vulnerability index (SVI), and COVID-19 community transmission level. The scoring of each data point ranged from 0–10, and outreach was prioritized to facilities with the highest overall scores. Successful referrals (resulting in a site visit) were compared to the SVI and healthcare emergency regional maps to determine whether the new outreach method reached more facilities in vulnerable communities. **Results:** Of 358 outreach attempts, IPRAT had a higher success rate with method 2 (6.9%) compared to method 1 (5.3%) and improved outreach in rural Michigan regions 7 and 8 (15% vs 3%). Site visits in counties with a high SVI rating with method 2 were 14.5% versus 10.6% using method 1. COVID-19 prevention referral success rates were higher (4.4% vs 3.1%) using method 2. **Conclusions:** The risk-assessment–based outreach method showed improvement in overall referral success rates among facilities in rural and higher-SVI counties. These communities tend to experience higher health disparities and poorer health outcomes. Incorporating the more nuanced data variables correlated with at-risk congregate-care settings receiving timelier outreach. The limitations of the study include sample size, period of data collected (2 years), and the complexity of objectively measuring equity.

**Disclosures:** None

